# Effects of omalizumab in children with asthma: A protocol for systematic review and meta-analysis

**DOI:** 10.1097/MD.0000000000026155

**Published:** 2021-06-04

**Authors:** Lu Chen, Yanping Chen

**Affiliations:** Department of Respiratory, Hunan Children's Hospital, Hunan, China.

**Keywords:** asthma, children, meta-analysis, omalizumab, protocol

## Abstract

**Background:**

It is still controversial in the current literature whether omalizumab is beneficial for children with asthma. Given that there is no high-quality meta-analysis to incorporate existing evidence, the purpose of this protocol is to design a systematic review and meta-analysis of the level I evidence to ascertain whether omalizumab is beneficial and safe for children with asthma.

**Methods:**

The systematic literature review is structured to adhere to Preferred Reporting Items for Systematic Reviews and Meta-analyses guidelines. The following search terms will be used in PUBMED, Scopus, EMBASE, and Cochrane Library databases on June, 2021, as the search algorithm: (omalizumab) AND (asthma) AND (children). The primary outcome is the long-term safety and tolerability of omalizumab. The other outcomes include asthma control, quality of life, use of asthma controller medications, and spirometry measurements and emergency room visits due to asthma, and serum trough concentrations of omalizumab, free and total immunoglobulin E measured. Review Manager software (v 5.3; Cochrane Collaboration) will be used for the meta-analysis.

**Results:**

The review will add to the existing literature by showing compelling evidence and improved guidance in clinic settings.

**Registration number:**

10.17605/OSF.IO/G6N3P.

## 1 Introduction

Childhood asthma is usually poorly controlled, possibly due to under-treatment with controller medications and poor inhalation techniques; however, despite the current availability of high-dose inhaled corticosteroids in addition to long-acting B2-agonists or leukotriene receptor antagonists, there is still a subset of children with poorly controlled asthma.^[1,2]^ Severe, hard-to-control asthma leads to a higher risk of asthma fever and impaired quality of life. Asthma exacerbation is often treated with systemic corticosteroids but may be associated with reduced bone mineral hyperplasia and an increased risk of osteoencephalopathy. Chronic use of high doses of inhaled corticosteroid has also been found to lead to inhibition of growth rate and adrenal function.^[3–5]^

Immunoglobulin E plays an important role in asthma and is often elevated in patients with severe disease and a history of atopic asthma. Omalizumab, a humanized monoclonal antibody against immunoglobulin E, was approved in 2003 for the treatment of asthma and has been shown to improve asthma symptoms, reduce the frequency of exacerbations, and reduce health care utilization and school absences. In addition, some studies have shown that omalizumab can improve lung function.^[6,7]^

It is still controversial in the current literature whether omalizumab is beneficial for children with asthma. Recent cohort studies and reviews have tried to resolve this issue, but have reached inconsistent conclusions.^[8–11]^ In addition, the literature on meta-analyses based only on randomized controlled trials (RCTs) is limited and many new RCTs have been published. Given that there is no high-quality meta-analysis to incorporate existing evidence, the purpose of this protocol is to design a systematic review and meta-analysis of the level I evidence to ascertain whether omalizumab is beneficial and safe for children with asthma.

## 2 Materials and methods

### 2.1 Search strategy

The systematic literature review is structured to adhere to Preferred Reporting Items for Systematic Reviews and Meta-analyses guidelines, which include requirements deemed essential for the transparent reporting of results. We will update our protocol for any changes in the entire research process if needed. The following search terms will be used in PUBMED, Scopus, EMBASE, and Cochrane Library databases on June, 2021, as the search algorithm: (omalizumab) AND (asthma) AND (children). Two searchers will independently draft and carry out the search strategy, and the third member will further complete it. References within included articles are reviewed to include articles that are not included within our literature search. The systematic review protocol has been registered on Open Science Framework registries. The registration number is 10.17605/OSF.IO/G6N3P. Since this study is on the basis of published or registered studies, ethical approval and informed consent of patients are not required.

### 2.2 Inclusion and exclusion criteria

Included studies are considered eligible if they met the population, intervention, comparator, outcomes, and study design criteria as follows:

- Population: children with asthma;
- Intervention: group with omalizumab;
- Comparator: group without omalizumab;
- Outcomes: The primary outcome is the long-term safety and tolerability of omalizumab. The other outcomes include asthma control, quality of life, use of asthma controller medications, and spirometry measurements and emergency room visits due to asthma, and serum trough concentrations of omalizumab, free and total immunoglobulin E measured.
- Study design: RCTs.

Exclusion criteria include observational studies, non-RCTs, review articles, studies with a sample size <50, and studies with insufficient outcome data.

### 2.3 Data extraction

Two independent authors will extract the following descriptive raw information from the selected studies: study characteristics such as author, study design, study language, publication year, mean follow-up period; patient demographic details such as number, average age, body mass index, and gender ratio; details of interventions, and outcome measures. The primary outcome is the long-term safety and tolerability of omalizumab. The other outcomes include asthma control, quality of life, use of asthma controller medications, and spirometry measurements and emergency room visits due to asthma, and serum trough concentrations of omalizumab, free and total immunoglobulin E measured. If the data are missing or cannot be extracted directly, we will contact the corresponding authors to ensure that the information is integrated. Otherwise, we calculate them with the guideline of Cochrane Handbook for Systematic Reviews of Interventions 5.1.0.

### 2.4 Data analysis

Review Manager software (v 5.3; Cochrane Collaboration) will be used for the meta-analysis. Extracted data are entered into Review Manager by the first independent author and checked by the second independent author. Risk ratio with a 95% confidence interval or standardized mean difference with 95% CI is assessed for dichotomous outcomes or continuous outcomes, respectively. The heterogeneity is assessed by using the Q test and *I*^*2*^ statistic. An *I*^*2*^ value of <25% is chosen to represent low heterogeneity and an *I*^*2*^ value of >75% to indicate high heterogeneity. All outcomes will be pooled on random-effect model. A *P* value of <.05 is considered to be statistically significant.

### 2.5 Risk of bias

The Cochrane risk of bias tool will be independently used to evaluate the risk of bias of included RCTs by 2 reviewers. The quality of RCTs will be assessed by using the following 7 items: random sequence generation, allocation concealment, blinding of participants and personnel, blinding of outcome assessment, incomplete outcome data, selective reporting, and other bias. Any controversy will be resolved by discussing with a third author to reach a final consensus.

## 3 Discussion

It is still controversial in the current literature whether omalizumab is beneficial for children with asthma. Recent cohort studies and reviews have tried to resolve this issue, but have reached inconsistent conclusions.^[8–11]^ In addition, the literature on meta-analyses based only on RCTs is limited and many new RCTs have been published. Given that there is no high-quality meta-analysis to incorporate existing evidence, the purpose of this protocol is to design a systematic review and meta-analysis of the level I evidence to ascertain whether omalizumab is beneficial and safe for children with asthma.

## Author contributions

**Conceptualization:** Yanping Chen.

**Data curation:** Lu Chen, Yanping Chen.

**Formal analysis:** Lu Chen, Yanping Chen.

**Funding acquisition:** Yanping Chen.

**Investigation:** Lu Chen, Yanping Chen.

**Methodology:** Lu Chen, Yanping Chen.

**Project administration:** Yanping Chen.

**Resources:** Yanping Chen.

**Software:** Lu Chen.

**Writing – original draft:** Lu Chen.

**Writing – review & editing:** Yanping Chen.
